# Assembly-promoting protein Munc18c stimulates SNARE-dependent membrane fusion through its SNARE-like peptide

**DOI:** 10.1016/j.jbc.2022.102470

**Published:** 2022-09-08

**Authors:** Furong Liu, Ruyue He, Min Zhu, Lin Zhou, Yinghui Liu, Haijia Yu

**Affiliations:** 1 Jiangsu Key Laboratory for Molecular and Medical Biotechnology, College of Life Sciences, Nanjing Normal University, Nanjing, China; 2 School of Chemistry and Bioengineering, Nanjing Normal University Taizhou College, Taizhou, China

**Keywords:** membrane fusion, SNARE, Munc18c, exocytosis, SM protein, CTD, C-terminal domain, FRET, fluorescence resonance energy transfer, MBP, maltose-binding protein, NBD, N-(7-nitro-2,1,3-benzoxadiazole-4-yl), SLP, SNARE-like peptide, SM, Sec1/Munc18, SNARE, soluble N-ethylmaleimide-sensitive factor attachment protein receptor, TCEP, Tris(2carboxyethyl) phosphine, TEV, tobacco etch virus, TMD, transmembrane domain

## Abstract

Intracellular vesicle fusion requires the soluble N-ethylmaleimide-sensitive factor attachment protein receptors (SNAREs) and their cognate Sec1/Munc18 (SM) proteins. How SM proteins act in concert with *trans*-SNARE complexes to promote membrane fusion remains incompletely understood. Munc18c, a broadly distributed SM protein, selectively regulates multiple exocytotic pathways, including GLUT4 exocytosis. Here, using an *in vitro* reconstituted system, we discovered a SNARE-like peptide (SLP), conserved in Munc18-1 of synaptic exocytosis, is crucial to the stimulatory activity of Munc18c in vesicle fusion. The direct stimulation of the SNARE-mediated fusion reaction by SLP further supported the essential role of this fragment. Interestingly, we found SLP strongly accelerates the membrane fusion rate when anchored to the target membrane but not the vesicle membrane, suggesting it primarily interacts with t-SNAREs *in cis* to drive fusion. Furthermore, we determined the SLP fragment is competitive with the full-length Munc18c protein and specific to the cognate v-SNARE isoforms, supporting how it could resemble Munc18c’s activity in membrane fusion. Together, our findings demonstrate that Munc18c facilitates SNARE-dependent membrane fusion through SLP, revealing that the t-SNARE-SLP binding mode might be a conserved mechanism for the stimulatory function of SM proteins in vesicle fusion.

Exocytosis is a process that delivers membrane proteins to the cell surface or releases cargoes to the extracellular matrix (1, 2). The fusion of exocytic vesicles and plasma membrane is regulated by the soluble N-ethylmaleimide-sensitive factor attachment protein receptors (SNAREs) and SNARE regulators. The central event of the fusion is that the target (t-) SNAREs pair with the vesicle (v-) SNARE to form a four-helix *trans*-SNARE complex (3, 4). N- to C- terminal SNARE zippering brings the two apposed membranes into a proximity that releases the free energy and initiates the fusion (5, 6, 7, 8, 9, 10, 11). In regulated exocytosis, the SNARE zippering is controlled by multiple regulators (12, 13, 14, 15). Significantly, the *trans*-SNARE complexes are unable to efficiently assemble unless activated by Sec1/Munc18 (SM) proteins (5, 16, 17, 18). SM proteins are evolutionarily conserved cytosolic proteins of 60 to 70 kDa that fold into an arch-shaped “clasp” structure (19). As the essential SNARE partners, SM proteins selectively recognize cognate v- and t-SNAREs and promote their assembly (17, 18, 20, 21, 22).

Insulin-stimulated glucose transporter 4 (GLUT4) exocytosis is critical in maintaining blood glucose homeostasis (23, 24, 25, 26). The defect of GLUT4 exocytosis ultimately leads to insulin resistance and type 2 diabetes (27, 28, 29). In GLUT4 exocytosis, the t-SNAREs are syntaxin4 and SNAP-23 (30, 31). While VAMP2 is the primary v-SNARE in GLUT4 SNARE-mediated vesicle fusion, VAMP3 and VAMP8 may play redundant or compensatory roles (32, 33, 34). Munc18c was identified as the predominant SM protein in GLUT4 exocytosis (35, 36, 37, 38). The imbalance of Munc18c is associated with insulin resistance and obesity (39, 40, 41). Our previous studies suggest that Munc18c is a positive regulatory factor promoting *trans*-SNARE zippering at the postdocking stage in GLUT4 SNARE-mediated vesicle fusion (17, 18). Unlike Munc18-1, a well-studied synaptic SM protein, the Munc18c–syntaxin4 dimer does not block the formation of the binary t-SNARE complex (17, 42, 43, 44). Munc18c and Munc18-1 recognize different regions on the SNARE complex, further suggesting the two SM proteins have conserved and divergent mechanisms in vesicle fusion (17). Therefore, although the mechanisms of Munc18-1 have been extensively studied, it remains unclear how Munc18c interacts with t- or v-SNAREs to regulate the SNARE zippering and vesicle fusion.

In synaptic vesicle fusion, domain 3a of Munc18-1 forms an extended helical structure upon SNARE binding, facilitating the SNARE assembly and vesicle priming (45, 46, 47, 48). Our recent study identified a SNARE-like peptide (SLP) in domain 3a of Munc18-1 that markedly accelerates synaptic SNARE-dependent liposome fusion (49). Synaptic SNAREs are known to possess specialized functions not found in other SNAREs. Thus, it is critical to determine whether the role of SLP is also crucial in another pathway. Interestingly, the sequence of SLP that contains heptad repeats of hydrophobic residues aligned with the v-SNARE C-terminal domain (CTD) is highly conserved in Munc18c (Fig. S1). However, whether the SLP of Munc18c has a conserved fusion-stimulating function in GLUT4 SNARE-driven membrane fusion is still uncertain.

In this work, we performed *in vitro* reconstitution assays to unravel the molecular mechanisms of Munc18c in SNARE-mediated fusion reaction. We observed that the conserved SLP in domain 3a is essential for the stimulatory activity of Munc18c in GLUT4 v- and t-SNARE-mediated liposome fusion. The short peptide fragment could resemble the stimulatory function of Munc18c in SNARE-dependent vesicle fusion. When we anchored SLP to the t-SNARE liposomes or added soluble SLP to the reactions, SLP strongly accelerated the fusion kinetics. In contrast, little change in the fusion rate was observed when SLP was reconstituted to the v-SNARE liposomes. While SLP specifically interacts with t-SNAREs, the cognate isoform of v-SNARE is required for its fusion stimulation, supporting the biological relevance of our study. Together, our findings disclose that Munc18c binds and activates t-SNAREs to accelerate GLUT4 SNARE-mediated fusion reaction primarily through its SLP.

## Results

### SLP is essential for the stimulatory function of Munc18c in reconstituted membrane fusion

**Figure 1:**
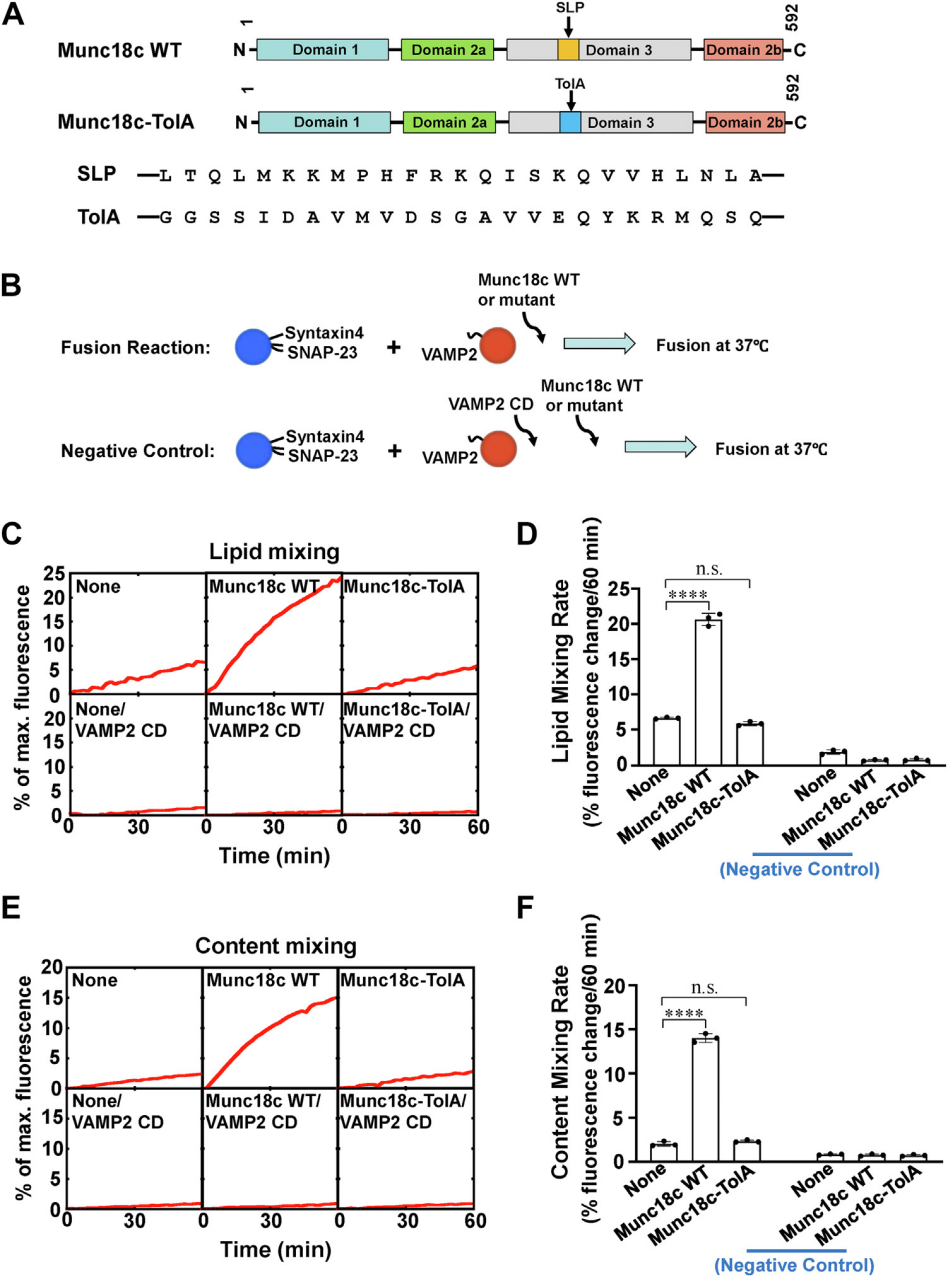
**SLP is indispensable for the stimulatory function of Munc18c in membrane fusion.***A*, *top*, diagrams of WT and mutant Munc18c proteins used in the liposome fusion assays. SLP corresponds to amino acids 327 to 351 of Munc18c. Munc18c-TolA refers to the replacement of the SLP with a bacterial TolA sequence. *Bottom*, sequences of SLP and TolA. *B*, illustration of the reconstituted liposome fusion procedures. *C*, lipid mixing of the reconstituted fusion reactions in the absence or presence of 5 μM Munc18c WT or Munc18c-TolA. Each fusion reaction contained 5 μM t-SNAREs, 1.5 μM v-SNARE, and 100 mg/ml Ficoll 70. Negative control 20 μM of VAMP2 CD was added at the beginning of the fusion reaction. *D*, lipid mixing rates of the reconstituted fusion reactions shown in (*C*). Data are presented as the percentage of fluorescence change per 60 min. Error bars indicate standard deviation. Data are presented as mean ± SD (n = 3 independent replicates). *p* Values were calculated using ordinary one-way ANOVA with Tukey’s multiple comparisons test. n.s., *p* > 0.05. ∗∗∗∗*p* < 0.0001. *E*, content mixing of the reconstituted fusion reactions in the absence or presence of 5 μM Munc18c or Munc18c-TolA proteins. Each fusion reaction contained 5 μM t-SNAREs, 1.5 μM v-SNARE, and 100 mg/ml Ficoll 70. Negative control 20 μM of VAMP2 CD was added at the beginning of the fusion reaction. *F*, content mixing rates of the reconstituted fusion reactions shown in (*E*). Data are presented as percentage of fluorescence change per 60 min. Error bars indicate standard deviation. Data are presented as mean ± SD (n = 3 independent replicates). *p* Values were calculated using ordinary one-way ANOVA with Tukey’s multiple comparisons test. n.s., *p* > 0.05. ∗∗∗∗*p* < 0.0001. CD, cytoplasmic domain; SLP, SNARE-like peptide; SNARE, soluble N-ethylmaleimide-sensitive factor attachment protein receptor.

To study whether the SLP of Munc18c plays a crucial role in membrane fusion, we replaced SLP with the N-terminal TolA, a nonrelated bacterial helix, to make the Munc18c-TolA chimeric protein (Fig. 1*A*). The circular dichroism spectra of WT Munc18c and Munc18c-TolA chimeric protein were similar (Fig. S2), suggesting that the overall folding of Munc18c protein was not altered by the SLP to TolA replacement. The lipid mixing and content mixing assays were then employed to monitor the fusion of v- and t-SNARE liposomes (Fig. 1*B*) (49, 50). In the fluorescence resonance energy transfer (FRET)–based lipid mixing assay, GLUT4 v- and t-SNAREs drove a basal level of lipid mixing, which was significantly enhanced by wildtype (WT) Munc18c (Fig. 1, *C* and *D*). We observed that the stimulatory activity of Munc18c was abrogated when SLP was replaced by TolA (Fig. 1, *C* and *D*).

In the content mixing assay, the soluble dye sulforhodamine B was encapsulated in the VAMP2 liposomes in which the fluorescence was self-quenched. The occurrence of fusion led to the dilution of sulforhodamine B so that the fluorescence self-quenching was relieved (17). Similarly, we observed that WT Munc18c rather than Munc18c-TolA chimera stimulated content mixing of the fusion reaction (Fig. 1, *E* and *F*). The lipid or content mixing was completely reduced to background levels by the dominant-negative inhibitor VAMP2 cytoplasmic domain, confirming that WT Munc18c and Munc18c-TolA chimera mediated liposome fusion through the regulation of SNARE assembly (Fig. 1, *C*–*F*). Together, these results demonstrate that SLP is crucial to the stimulatory activity of Munc18c in GLUT4 SNARE-mediated membrane fusion.

### Munc18c SLP directly stimulates SNARE-dependent membrane fusion

**Figure 2:**
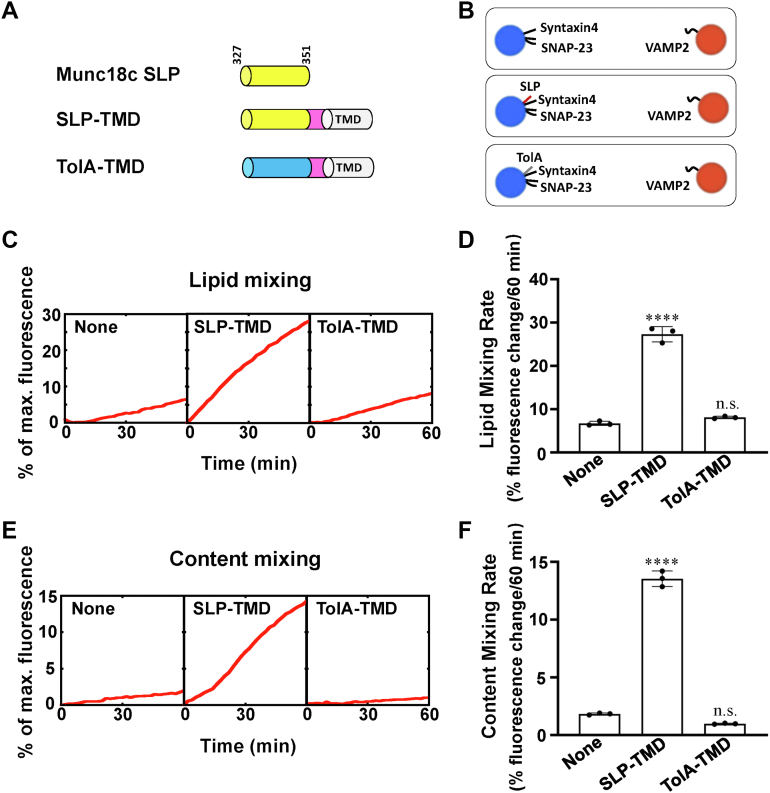
**SLP activates SNARE-mediated membrane fusion when it is anchored to the membrane.***A*, diagrams of Munc18c SLP and TolA with an engineered transmembrane domain (TMD, from VAMP2). *B*, illustration of the liposome fusion pairs. *C*, lipid mixing of the reconstituted fusion reactions containing 5 μM t-SNAREs, 1.5 μM WT VAMP2, 100 mg/ml Ficoll 70, and 5 μM of the indicated chimeric protein. The fusion reactions were measured by a FRET-based lipid mixing assay. *D*, lipid mixing rates of the reconstituted fusion reactions shown in (*C*). Data are presented as the percentage of fluorescence change per 60 min. Error bars indicate standard deviation. Data are presented as mean ± SD (n = 3 independent replicates). *p* Values were calculated using ordinary one-way ANOVA with Tukey’s multiple comparisons test. n.s., *p* > 0.05. ∗∗∗∗*p* < 0.0001. *E*, content mixing of the reconstituted fusion reactions described in (*C*). *F*, content mixing rates of the reconstituted fusion reactions shown in (*E*). Data are presented as the percentage of fluorescence change per 60 min. Error bars indicate standard deviation. Data are presented as mean ± SD (n = 3 independent replicates). *p* Values were calculated using ordinary one-way ANOVA with Tukey’s multiple comparisons test. n.s., *p* > 0.05. ∗∗∗∗*p* < 0.0001. FRET, fluorescence resonance energy transfer; SLP, SNARE-like peptide; SNARE, soluble N-ethylmaleimide-sensitive factor attachment protein receptor.

Next, we examined how SLP regulates the GLUT4 SNARE-mediated membrane fusion reaction. To strengthen the activity, we created the SLP–transmembrane domain (TMD) fusion protein in which SLP was combined with an engineered membrane anchor (Fig. 2*A*) (49). When we reconstituted SLP to t-SNARE liposomes through the membrane anchor, it markedly stimulated the SNARE-dependent liposome fusion (Fig. 2, *B*–*F*). The fusion stimulation depended on the concentration of membrane-anchored SLP in the reaction (Fig. S3). On the contrary, the membrane-anchored TolA helix was unable to accelerate the fusion kinetics (Fig. 2, *B*–*F*). Moreover, the change of the membrane anchor does not affect the stimulatory activity of SLP, excluding the influence of the transmembrane domain on the fusion activity (Fig. S4).

**Figure 3:**
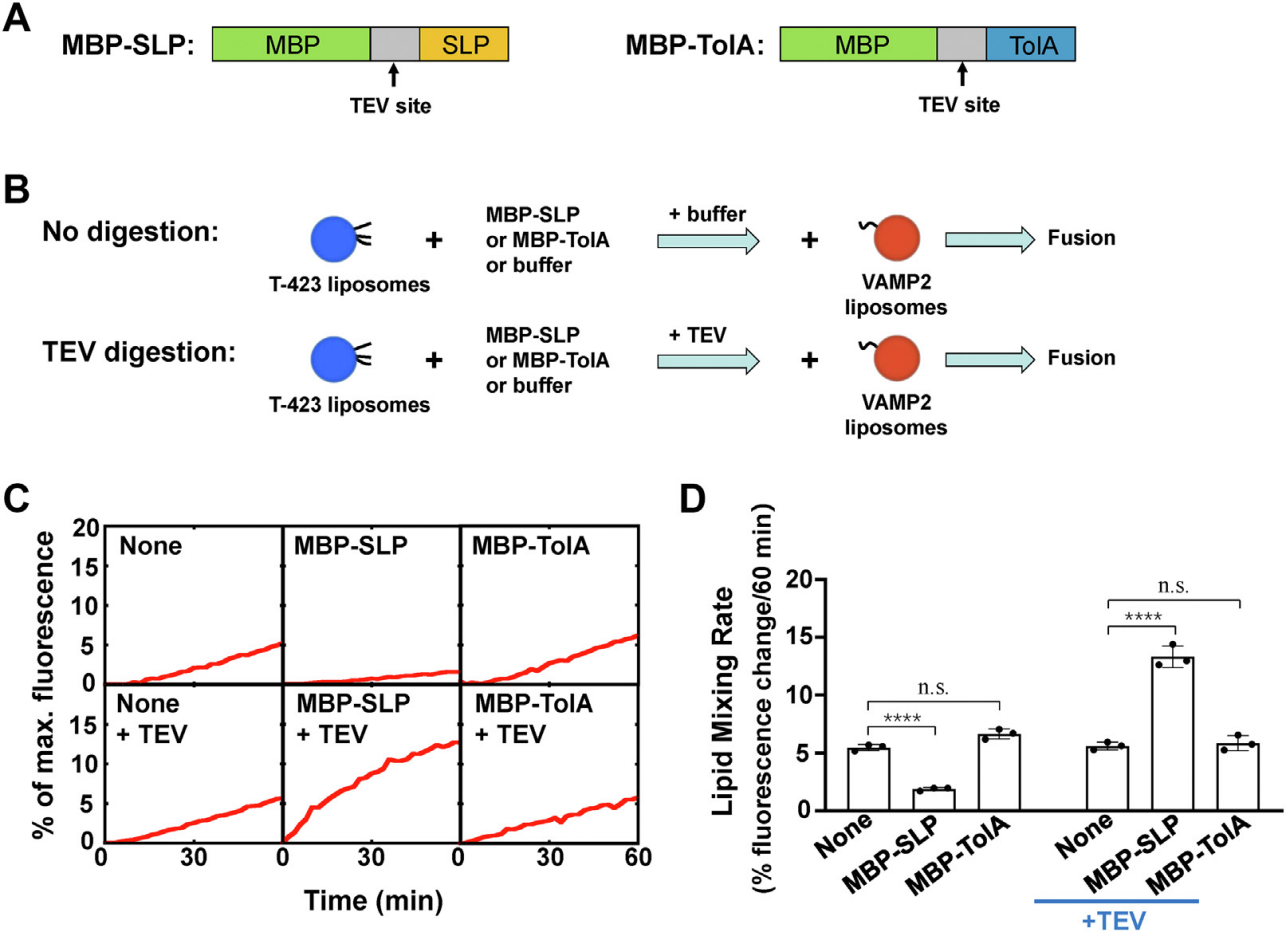
**Munc18c SLP stimulates SNARE-dependent membrane fusion in solution.***A*, diagram of the MBP-SLP and MBP-TolA. A TEV cleavage site (ENLYFQG) was inserted between MBP and SLP. *B*, illustration of the experimental procedure of the reconstituted fusion reactions. *C*, activation of SNARE-mediated fusion reactions by soluble SLP. The t-SNARE liposomes containing syntaxin4 and SNAP-23 were incubated with or without 5 μM indicated protein in the absence or presence of TEV protease at 37 °C. After 30 min, VAMP2 liposomes were introduced to initiate fusion. The fusion reactions were measured by a FRET-based lipid mixing assay. *D*, lipid mixing rates of the reconstituted fusion reactions shown in (*C*). Data are presented as the percentage of fluorescence change per 60 min. Error bars indicate standard deviation. Data are presented as mean ± SD (n = 3 independent replicates). *p* Values were calculated using two-way ANOVA with Tukey’s multiple comparisons test. n.s., *p* > 0.05. ∗∗∗∗*p* < 0.0001. FRET, fluorescence resonance energy transfer; MBP, maltose-binding protein; SLP, SNARE-like peptide; SNARE, soluble N-ethylmaleimide-sensitive factor attachment protein receptor; TEV, tobacco etch virus.

We then purified maltose-binding protein (MBP)–tagged SLP to test the effect of soluble SLP on membrane fusion. A tobacco etch virus (TEV) protease cleavage site was introduced between MBP and SLP (Fig. 3*A*). Unexpectedly, we observed that MBP-SLP efficiently blocked the SNARE-mediated liposome fusion (Fig. 3, *B*–*D*). After removing MBP by TEV protease digestion, the fusion kinetics was dramatically accelerated (Fig. 3, *B*–*D*). The concentration-dependent activities of MBP-SLP suggest that the fusion inhibition or stimulation is explicitly mediated by intact or TEV-digested MBP-SLP (Fig. S5). In the control experiments, MBP-TolA fusion protein drove a liposome fusion at a level comparable to basal fusion, whenever with or without TEV protease digestion (Fig. 3, *C* and *D*). We postulated that the large MBP protein might hinder the t- and v- SNARE assembly after SLP-SNARE interaction, resulting in a fusion inhibition. Indeed, when MBP was removed, SLP became active and accelerated the fusion reaction (Fig. 3, *C* and *D*). All the above experimental results demonstrated that SLP was capable of stimulating SNARE-dependent membrane fusion.

### Munc18c promotes SNARE-dependent membrane fusion through SLP

**Figure 4:**
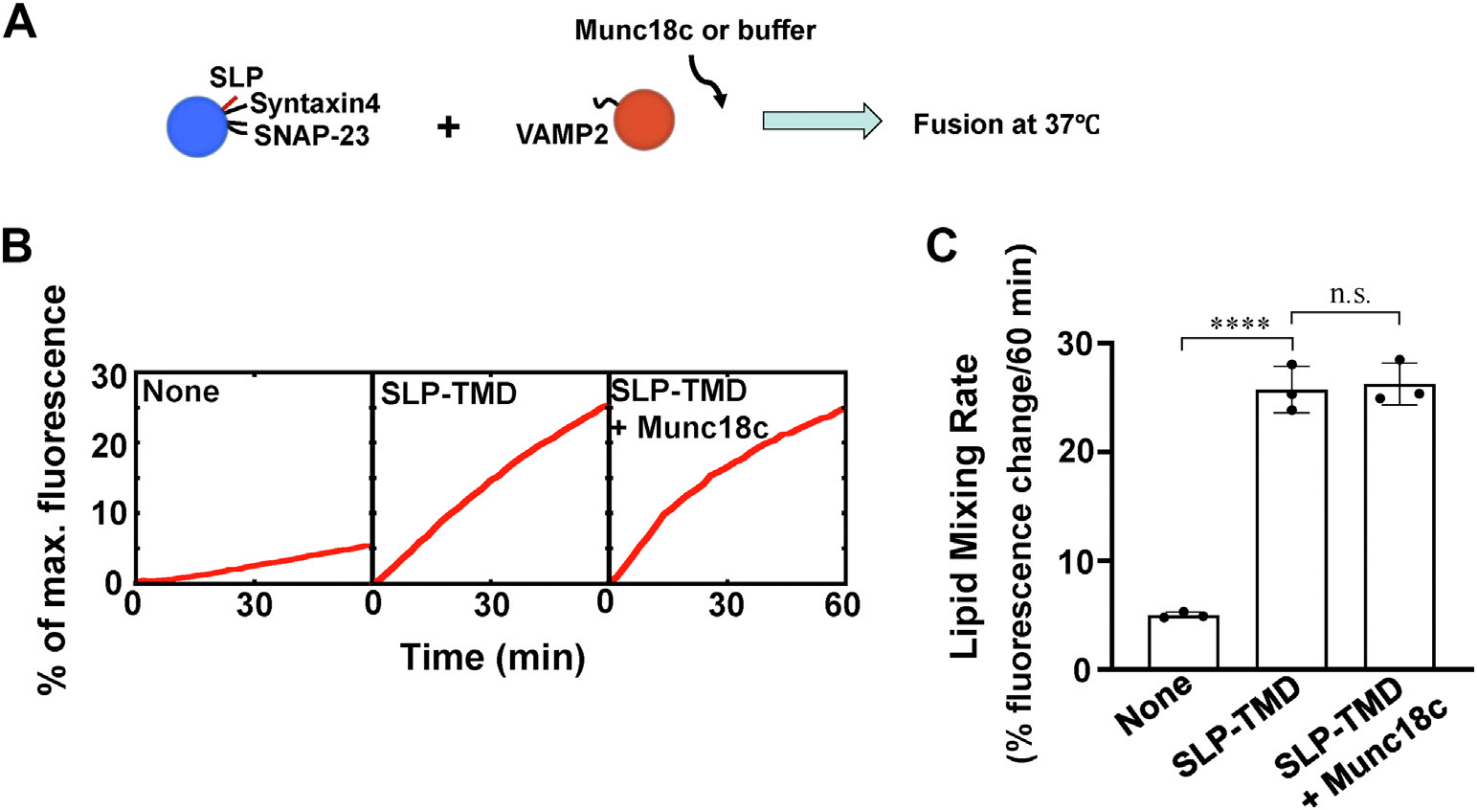
**Munc18c activates membrane fusion through SLP.***A*, diagram illustrating the experimental procedure of the reconstituted fusion reactions. *B*, reconstituted liposome fusion reactions in the absence or presence of 5 μM Munc18c. Each fusion reaction contained 5 μM t-SNAREs, 1.5 μM v-SNARE, and 100 mg/ml Ficoll 70. The fusion reactions were measured by a FRET-based lipid mixing assay. *C*, lipid mixing rates of the reconstituted fusion reactions shown in (*B*). Data are presented as the percentage of fluorescence change per 60 min. Error bars indicate standard deviation. Data are presented as mean ± SD (n = 3 independent replicates). *p* Values were calculated using ordinary one-way ANOVA with Tukey’s multiple comparisons test. n.s., *p* > 0.05. ∗∗∗∗*p* < 0.0001. FRET, fluorescence resonance energy transfer; SLP, SNARE-like peptide; SNARE, soluble N-ethylmaleimide-sensitive factor attachment protein receptor.

We then attempt to delineate how Munc18c regulates membrane fusion in the presence of SLP (Fig. 4*A*). The fusion reactions containing membrane-anchored SLP were not further stimulated by Munc18c, indicating SLP is competitive with WT Munc18c in the membrane fusion (Fig. 4, *B* and *C*). These data support that Munc18c promotes SNARE-dependent membrane fusion primarily through SLP, similar to Munc18-1 in synaptic vesicle fusion (49).

### SLP interacts with t-SNAREs *in cis* to activate SNARE-dependent membrane fusion

**Figure 5:**
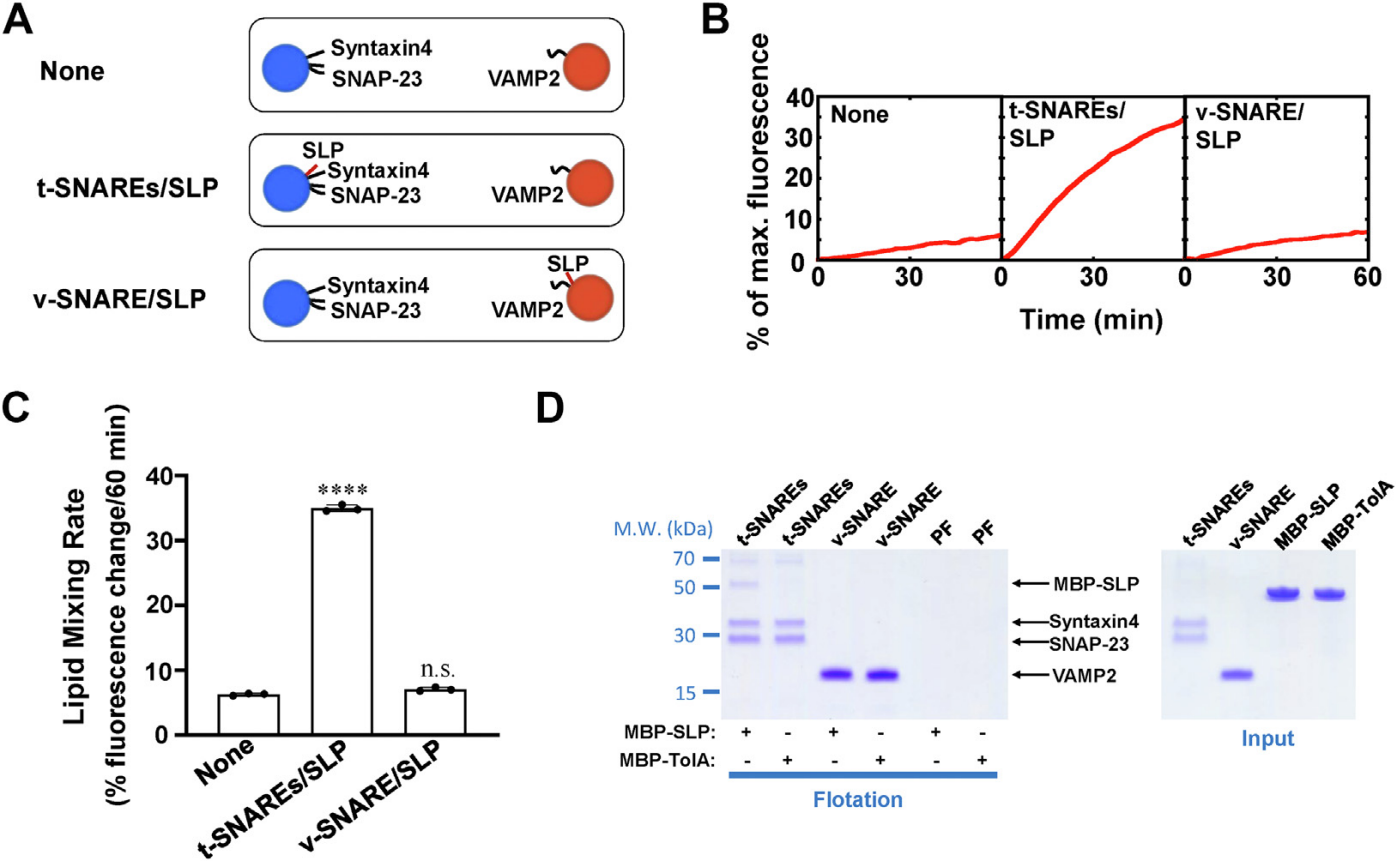
**SLP interacts with t-SNAREs to activate membrane fusion in the *cis*-configuration.***A*, illustration of the liposome fusion pairs. *B*, lipid mixing of the reconstituted fusion reactions containing 5 μM t-SNAREs, 1.5 μM v-SNARE, 100 mg/ml Ficoll 70. The fusion reactions were measured by a FRET-based lipid mixing assay. *C*, lipid mixing rates of the reconstituted fusion reactions shown in (*B*). Data are presented as the percentage of fluorescence change per 60 min. Error bars indicate standard deviation. Data are presented as mean ± SD (n = 3 independent replicates). *p* Values were calculated using ordinary one-way ANOVA with Tukey’s multiple comparisons test. n.s., *p* > 0.05. ∗∗∗∗*p* < 0.0001. *D*, Coomassie blue–stained SDS/PAGE gel showing the binding of Munc18c SLP to t-SNARE liposomes containing syntaxin4 and SNAP-23. The v-SNARE liposome was composed of VAMP2. The liposomes were prepared with 100% phosphatidylcholine (PC). Each binding reaction contained 5 μM SNAREs and 5 μM MBP-tagged peptide. FRET, fluorescence resonance energy transfer; MBP, maltose-binding protein; PF, protein-free; SLP, SNARE-like peptide; SNARE, soluble N-ethylmaleimide-sensitive factor attachment protein receptor.

Previous studies suggested the fusion stimulation of SM protein requires SNAREs in the restricted topology (51, 52). We then tested whether the ability of SLP to promote membrane fusion is topology dependent. SLP was reassembled into t- or v-SNARE liposomes by the membrane anchor (Fig. 5*A*). Interestingly, SLP had little effect on the kinetics of the SNARE-mediated liposome fusion when it was anchored to v-SNARE liposomes, indicating the activation of SLP requires it to interact with t-SNAREs *in cis* but not *in trans* (Fig. 5, *B* and *C*). In a liposome co-flotation assay, MBP-SLP was specifically bound to t-SNARE liposomes containing syntaxin4 and SNAP-23 but not VAMP2 or protein-free liposomes (Fig. 5, *D* and *E*). Previous studies showed syntaxin4 N-peptide interacts with domain 1 of Munc18c (53). We then removed the N-terminal domain from syntaxin4 (syntaxin4ΔN) to study whether SLP mediated Munc18c binding to the SNARE motifs (core domains) of t-SNAREs. Our results showed that WT Munc18c but not Munc18c-TolA interacted with t-SNARE liposomes containing syntaxin4ΔN and SNAP-23, suggesting Munc18c can bind to the SNARE motifs of t-SNAREs through SLP (Fig. S6). These data demonstrated that SLP stimulates membrane fusion by interacting with t-SNAREs at a *cis*-configuration.

### The stimulatory activity of Munc18c SLP is specific to its cognate v-SNARE isoforms

**Figure 6:**
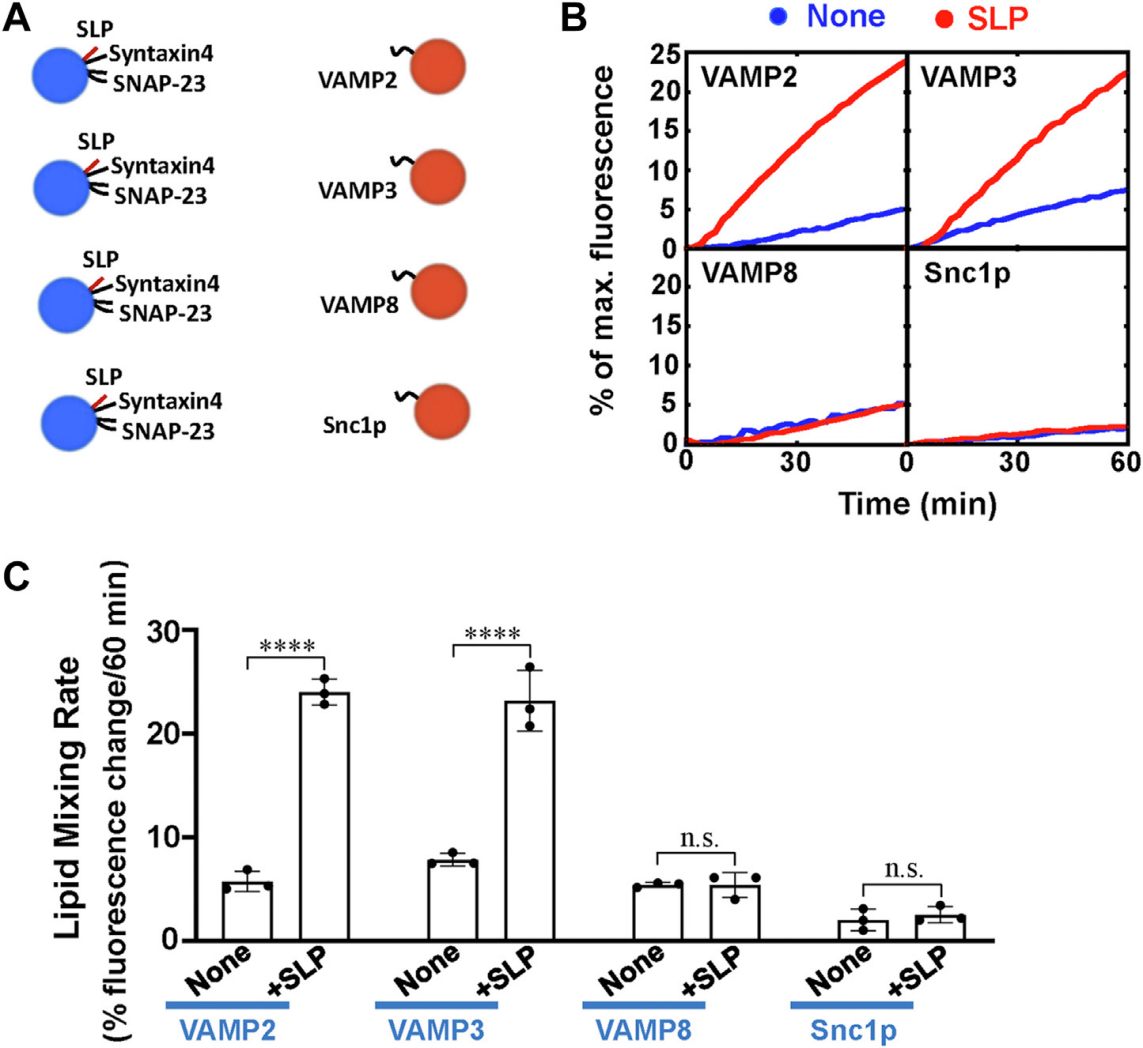
**The stimulatory function of SLP is sensitive to v-SNARE isoforms.***A*, illustration of the liposome fusion pairs. *B*, lipid mixing of the reconstituted fusion reactions containing 5 μM t-SNAREs, 1.5 μM v-SNARE, and 100 mg/ml Ficoll 70. The fusion reactions were measured by a FRET-based lipid mixing assay. *C*, lipid mixing rates of the reconstituted fusion reactions shown in (*B*). Data are presented as the percentage of fluorescence change per 60 min. Error bars indicate standard deviation. Data are presented as mean ± SD (n = 3 independent replicates). *p* Values were calculated using two-way ANOVA with Tukey’s multiple comparisons test. n.s., *p* > 0.05. ∗∗∗∗*p* < 0.0001. FRET, fluorescence resonance energy transfer; SLP, SNARE-like peptide; SNARE, soluble N-ethylmaleimide-sensitive factor attachment protein receptor.

It has been well defined that SM proteins only stimulate their cognate SNAREs-driven fusion reactions, reflecting the precise intracellular vesicle transport (54). Our previous studies showed although Munc18c is broadly distributed and recognizes a series of v-SNARE isoforms, it could not stimulate the fusion reactions containing VAMP8 or yeast v-SNAREs (17). We then tested whether SLP has the selectivity for v-SNARE isoforms in our reconstitution system. The t-SNARE liposomes containing syntaxin4/SNAP-23 and SLP were directed to fuse with liposomes containing diverse v-SNARE isoforms (Fig. 6*A*). While the cognate VAMP2 and VAMP3 supported the stimulatory activity, SLP was unable to stimulate the fusion reactions reconstituted with VAMP8 or yeast Snc1p (Fig. 6, *B* and *C*). These data reveal that SLP retains the intrinsic cognate v-SNARE specificity of Munc18c, supporting the biological relevance of the findings.

## Discussion

Impairment in insulin-stimulated GLUT4 exocytosis is a hallmark of insulin resistance and type 2 diabetes. Extensive studies have been performed on SNARE-mediated GLUT4 exocytosis. A variety of SNARE-binding factors were identified to control the assembly of the *trans*-SNARE complex, which drives membrane fusion between the GLUT4 storage vesicles and plasma membrane (2, 25, 55, 56, 57, 58, 59, 60). It has been established that the SM protein Munc18c positively regulates GLUT4 SNARE-mediated vesicle fusion, but the molecular mechanism remains less understood (17, 18, 37, 39, 61). Previous studies suggest that SM proteins take conserved and divergent mechanisms in SNARE-mediated fusion reactions (16, 17, 44, 62, 63). Therefore, it is worth examining how Munc18c regulates its cognate t- and v-SNARE-driven membrane fusion.

Membrane fusion is initiated when v- and t-SNAREs zipper into the *trans*-SNARE complex (64, 65). The N-terminal domains of the SNARE motifs pair, restructuring the t-SNAREs and setting the stage for the subsequent zippering of the CTDs (5, 15, 66, 67). However, SNAREs may not release their full fusion potential due to conformational constraints, which require SM proteins to facilitate the process (15, 67). Correspondingly, our previous studies showed that SM protein accelerates membrane fusion by acting on the SNARE CTD-mediated *trans*-SNARE assembly stage (17, 18, 56, 57). How the conformational constraint is removed by Munc18c is not fully understood. In this study, we characterized the SLP of Munc18c in the defined reconstitution system. We found that SLP is indispensable for Munc18c's stimulatory activity in fusion. SLP binds to t-SNAREs like v-SNARE CTD (Vc peptide) and efficiently accelerates SNARE-dependent membrane fusion (68, 69).

When we localized SLP to the vicinity of the t-SNAREs by an engineered transmembrane domain, it could resemble the activity of WT Munc18c protein in membrane fusion. The artificial membrane anchor may facilitate the recruitment of SLP to SNAREs. SLP failed to accelerate the membrane fusion rate when it was anchored to the vesicle membrane, indicating a requirement of *cis*-configuration. Our findings suggest that the stimulatory activity of SLP needs it to arrive at the nearby region of t-SNAREs with proper orientation. While the membrane-anchored Munc18c SLP fragment could stimulate membrane fusion, it is still worth knowing how the non-membrane-anchored SLP fragment acts on the fusion kinetics. Instead of stimulation, MBP-SLP blocked SNARE-dependent membrane fusion like a brake. Since MBP could be located nearby the SNARE motifs by SLP-t-SNARE interaction, it is reasonable that the large MBP protein impedes the next step of SNARE assembly. Actually, when MBP was removed, SLP turned to facilitate the membrane fusion, supporting the inhibitory role of the MBP protein. These results demonstrate that Munc18c SLP alone can stimulate SNARE-dependent membrane fusion directly.

Munc18-facilitated SNARE zippering is a conserved and highly dynamic process. The structure and folding dynamics of the t-SNARE complex are critical for the ternary SNARE assembly (49, 68). SLP has multiple binding modes during SNARE zippering. In addition to the t-SNARE complex, SLP and its adjacent regions can also recognize v-SNARE, facilitating the formation of the syntaxin–Munc18–VAMP template complex (16, 22, 47, 70, 71, 72). It was suggested that the physiological SNARE assemblies might start from Munc18-bound syntaxins (42, 44, 72). Then, Munc18 interacts with v-SNARE to form a syntaxin–Munc18–VAMP template complex, which may be universally conserved for Munc18-chaperoned SNARE assembly (16, 70, 71, 72). The template complex guides the formation of a partially zippered *trans*-SNARE complex, whereas Munc18 SLP turns to interact with the CTDs of the intermediate t-SNARE complex. The t-SNARE–SLP complex restructures t-SNAREs into a conformation suitable for complete zippering with v-SNARE, a mechanism similar to Vc peptide-stimulated fusion (49, 68).

While Munc18c uses its SLP to stimulate fusion, other domains may play additional functions, for example, maintaining the conformation and orientation of SLP. Beyond the direct regulation of *trans*-SNARE assembly, Munc18c has multiple other identified functions. Munc18c binds to syntaxin4, which facilitates its location at the exocytotic sites (42, 53, 73, 74). It also interacts with other regulatory factors, such as Doc2b, to cooperatively regulate GLUT4 exocytosis (75, 76). Furthermore, Munc18c may modulate GLUT4 exocytosis under the control of insulin signaling through tyrosine phosphorylation (73, 77).

In summary, our findings demonstrated that Munc18c interacts with t-SNAREs through its SLP and facilitates SNARE-dependent membrane fusion. Although Munc18-1 and Munc18c recognize distinct SNAREs and have divergent binding modes with their individual cognate SNAREs, they share the conserved mechanism through the action of SLP in stimulating vesicle fusion.

## Experimental procedures

### Protein expression and purification

Recombinant t- and v-SNARE proteins were expressed in *E. coli* and purified by affinity chromatography (21). GLUT4 exocytic t-SNAREs, comprised of the untagged syntaxin-4 and the His_6_-tagged SNAP-23, were expressed using the same procedure as previously described (17, 18). Full-length v-SNAREs were expressed similarly as previously described and contained no extra residues after the tags were proteolytically removed by SUMO protease (17, 18). SNAREs were stored in a buffer containing 25 mM Hepes (pH 7.4), 400 mM KCl, 1% n-octyl-β-D-glucoside, 10% (vol/vol) glycerol, and 0.5 mM Tris(2carboxyethyl) phosphine (TCEP). MBP-tagged SLP (aa. 327–351 of Munc18c) and TolA (aa. 1–25) were expressed in *E. coli* and purified by nickel affinity chromatography. The soluble proteins were stored in a buffer containing 25 mM Hepes (pH 7.4), 150 mM KCl, 10% (vol/vol) glycerol, and 0.5 mM TCEP.

Recombinant untagged Munc18c protein was produced in Sf9 insect cells using baculovirus infection (17). The full-length mouse Munc18c gene was subcloned into the baculovirus transfer vector pFastBac to generate a construct encoding a His_6_–Munc18c fusion protein separated by a TEV protease cleavage site. Munc18c proteins were purified from the Sf9 cells by nickel affinity as previously described (17). The His_6_ tag was removed from Munc18c by TEV protease, and the protein was subsequently dialyzed overnight against a storage buffer (25 mM Hepes [pH 7.4], 150 mM KCl, 10% glycerol, and 0.5 mM TCEP). Munc18c-TolA mutant was generated by site-directed mutagenesis and purified similarly to the WT protein.

The membrane-anchored SLP or TolA was expressed in *E. coli* and purified in a similar way as v-SNAREs. Sequences of chimeric proteins used in this work are listed below: (1) SLP-TMD (SLP is derived from amino acids 327–351 of mouse Munc18c and highlighted in bold, and TMD is derived from the transmembrane domain of VAMP2): **LTQLMKKMPHFRKQISKQVVHLNLA**KRKYWWKNLKMMIILGVICAIILIIIIVYFST. (2) TolA-TMD (the amino acids 1–25 of bacterial TolA sequence is shown in bold, and TMD is derived from the transmembrane domain of VAMP2): **GGSSIDAVMVDSGAVVEQYKRMQSQ**KRKYWWKNLKMMIILGVICAIILIIIIVYFST. (3) SLP-TMD∗ (SLP is derived from amino acids 327–351 of mouse Munc18c and highlighted in bold, and TMD is derived from the transmembrane domain of syntaxin4): **LTQLMKKMPHFRKQISKQVVHLNLA**IALENQKKARKKKVMIAICVSVTVLILAVIIGITITVG. (4) TolA-TMD∗ (the amino acids 1–25 of bacterial TolA sequence is shown in bold, and TMD is derived from the transmembrane domain of syntaxin4): **GGSSIDAVMVDSGAVVEQYKRMQSQ**IALENQKKARKKKVMIAICVSVTVLILAVIIGITITVG.

### Proteoliposome preparation

All lipids used in this work were acquired from Avanti Polar Lipids. For t-SNARE reconstitution, 1-palmitoyl-2-oleoyl-sn-glycero-3phosphocholine, 1-palmitoyl-2-oleoyl-sn-glycero-3-phosphoethanolamine, 1-palmitoyl-2-oleoyl-sn-glycero-3-phosphoserine, and cholesterol were mixed in a molar ratio of 60:20:10:10. To prepare v-SNARE liposomes, 1-palmitoyl-2-oleoyl-sn-glycero-3phosphocholine, 1-palmitoyl-2-oleoyl-sn-glycero-3-phosphoethanolamine, 1-palmitoyl-2-oleoyl-sn-glycero-3-phosphoserine, cholesterol, N-(7-nitro-2,1,3-benzoxadiazole-4-yl) (NBD)-1,2-dipalmitoyl phosphatidylethanolamine, and N(Lissamine rhodamine B sulfonyl)-1,2-dipalmitoyl phosphatidylethanolamine were mixed at a molar ratio of 60:17:10:10:1.5:1.5. SNARE proteoliposomes were prepared by detergent dilution and isolated on a Nycodenz density gradient flotation (49, 78). Detergent was removed by overnight dialysis using Novagen dialysis tubes against the reconstitution buffer (25 mM Hepes [pH 7.4], 100 mM KCl, 10% [vol/vol] glycerol, and 1 mM DTT). To prepare sulforhodamine-loaded liposomes, SNARE liposomes were reconstituted in the presence of 50 mM sulforhodamine B (Sigma). Free dye was removed by overnight dialysis, followed by liposome flotation on a Nycodenz gradient. The protein-to-lipid ratio was at 1:200 for v-SNAREs and at 1:500 for t-SNARE liposomes.

### Liposome lipid and content mixing assays

A standard liposome fusion reaction contained 5 μM t SNAREs, 1.5 μM v-SNARE, and 100 mg/ml of the macromolecular crowding agent Ficoll 70 and was conducted in a 96-well microplate at 37 °C. The fusion reactions were carried out in the reaction buffer (25 mM Hepes [pH 7.4], 50 mM KCl, and 1 mM DTT). In FRET-based lipid mixing assays, v-SNARE liposomes containing NBD-lipids quenched by rhodamine-lipids were directed to fuse with unlabeled t-SNARE liposomes. Increase in NBD-fluorescence at 538 nm (excitation 460 nm) was measured every 2 min in a BioTek Synergy HT microplate reader. At the end of the reaction, 10 μl of 10% 3-[(3-cholamidopropyl)dimethylammonio]- 2-hydroxy-1-propanesulfonic acid (CHAPSO) was added to the liposomes. For content mixing assays, unlabeled t-SNARE liposomes were directed to fuse with sulforhodamine B-loaded v-SNARE liposomes. The sulforhodamine B fluorescence (excitation: 565; emission: 585 nm) was measured every 2 min. At the end of the reaction, 10 μl of 10% CHAPSO was added to each sample. Fusion data were presented as the percentage of maximum fluorescence change. Full accounting of statistical significance was included for each dataset based on at least three independent experiments.

### Liposome co-flotation assay

The binding of soluble factors with membranes was examined using a liposome co-flotation assay, as we previously described (17, 49). MBP-tagged SLP or TolA was individually incubated with protein-free liposomes, t-SNARE liposomes containing syntaxin4 and SNAP23, or VAMP2 liposomes at 4 °C with gentle agitation. An equal volume (150 μl) of 80% Nycodenz (wt/vol) in the reconstitution buffer was added after 1 h, and the mixture was transferred to 5 × 41-mm centrifuge tubes. The liposomes were overlaid with 200 μl each of 35% and 30% Nycodenz and then with 20 μl of reconstitution buffer on the top. The gradients were centrifuged for 4 h at 48,000 rpm in a Beckman SW55 rotor. Liposome samples were collected from the 0/30% Nycodenz interface (2 × 20 μl) and analyzed by SDS-PAGE.

### Statistical analysis

All data were presented as the mean ± SD and were analyzed using GraphPad Prism 8.0.2 software for Windows. Statistical significance was calculated using one-way ANOVA or two-way ANOVA, and *p*-Value <0.05 was considered statistically significant.

## Data availability

All data presented are contained within the main manuscript and supporting information.

## Supporting information

This article contains supporting information.

## Conflict of interest

The authors declare that they have no conflicts of interest with the contents of this article.
