# Supplementary material for: Assembly-promoting protein Munc18c stimulates SNARE-dependent membrane fusion through its SNARE-like peptide
Source: J Biol Chem. 2022 Sep 8;298(10):102470. doi: 10.1016/j.jbc.2022.102470 (PMC9547204; doi:10.1016/j.jbc.2022.102470)
Supplement: Supplemental Figures S1–S6 [file mmc1.pdf]

Assembly-promoting protein Munc18c stimulates SNARE-dependent membrane fusion through its SNARE-like peptide

Furong Liu[1], Ruyue He[1], Min Zhu[1], Lin Zhou[2], Yinghui Liu[1*], and Haijia Yu[1*]

[1]Jiangsu Key Laboratory for Molecular and Medical Biotechnology, College of Life Sciences, Nanjing Normal University, Nanjing, 210023, China.

[2]School of Chemistry and Bioengineering, Nanjing Normal University Taizhou College, Taizhou, 225300, China.

*Correspondence: yinghuiliu@njnu.edu.cn (Y.L.); yuhaijia@njnu.edu.cn (H.Y.)

This SI file contains: Supplemental Figures 1 to 6.

	+1 *	+2 *	+3 *	+4 *	+5 *	+6 *	+7 *	+8 *
VAMP2 (aa.60-84):	**L**SE	**L**DDR	**A**DA	**L**QAG	**A**SQ	**F**ETS	**A**AK	**L**
Rat Munc18-1 (aa.327-351):	**L**SQ	**M**LKK	**M**PQ	**Y**QKE	**L**SK	**Y**STH	**L**HL	**A**
Mouse Munc18c (aa.327-351):	**L**TQ	**L**MKK	**M**PH	**F**RKQ	**I**SK	**Q**VVH	**L**NL	**A**

Figure S1. The sequence of SLP is conserved in Munc18c. Sequences of VAMP2 CTD and the SLPs derived from Munc18-1 and Munc18c were shown.

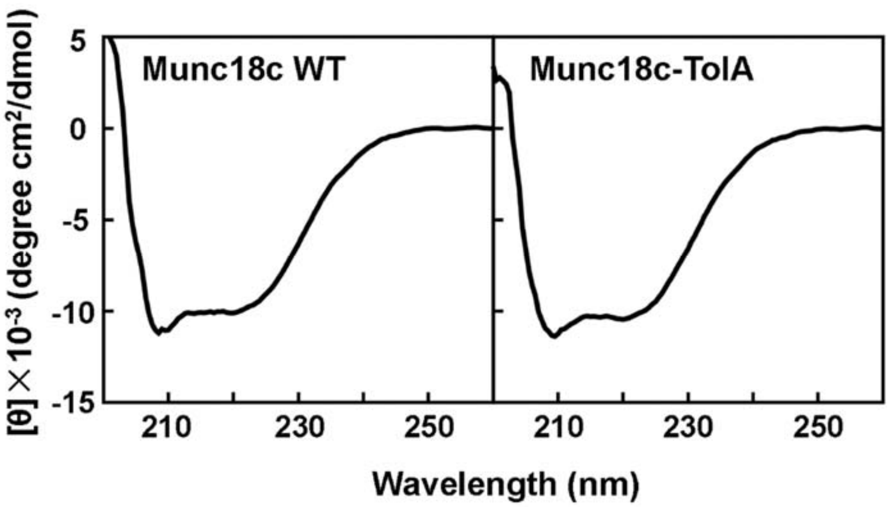

Figure S2. Circular dichroism spectroscopic analysis of WT and mutant Munc18c proteins. The circular dichroism spectra were measured using a Chirascan spectropolarimeter equipped with a 1 mm quartz cell. The readings were made at 0.5 nm intervals, and each data point represented the average of six scans at a speed of 50 nm/min over the wavelength range of 200 nm to 260 nm. The data were converted into mean residue weighted molar ellipticity using the following equation: $[\theta]_{MRW} = (100 \times \theta)/Cnl$, where C is the protein concentration (mM), θ is the measured ellipticity (milli-degree), n is the number of residues, and l is the path length (cm).

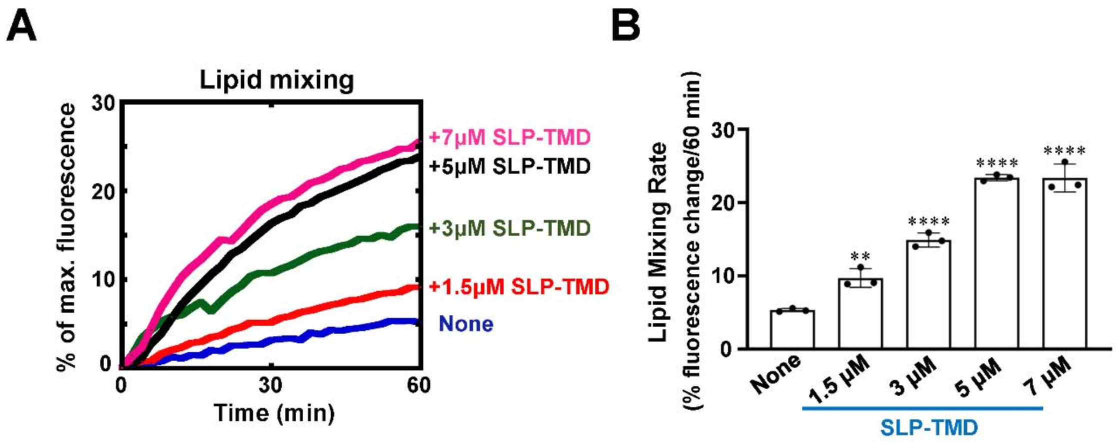

Figure S3. The membrane-anchored SLP stimulates SNARE-mediated membrane fusion in a concentration-dependent manner. (A) Lipid mixing of the reconstituted fusion reactions containing 5 μM t-SNAREs, 1.5 μM WT VAMP2, 100 mg/mL Ficoll 70, and the indicated concentrations of membrane-anchored SLP. The fusion reactions were measured by a FRET-based lipid mixing assay. **(B)** Lipid mixing rates of the reconstituted fusion reactions shown in A. Data are presented as percentage of fluorescence change per 60 min. Error bars indicate standard deviation. Data are presented as mean ± SD (n = 3 independent replicates). p Values were calculated using ordinary one-way ANOVA with Tukey's multiple comparisons test. $^{**}p < 0.01$. $^{****}p < 0.0001$.

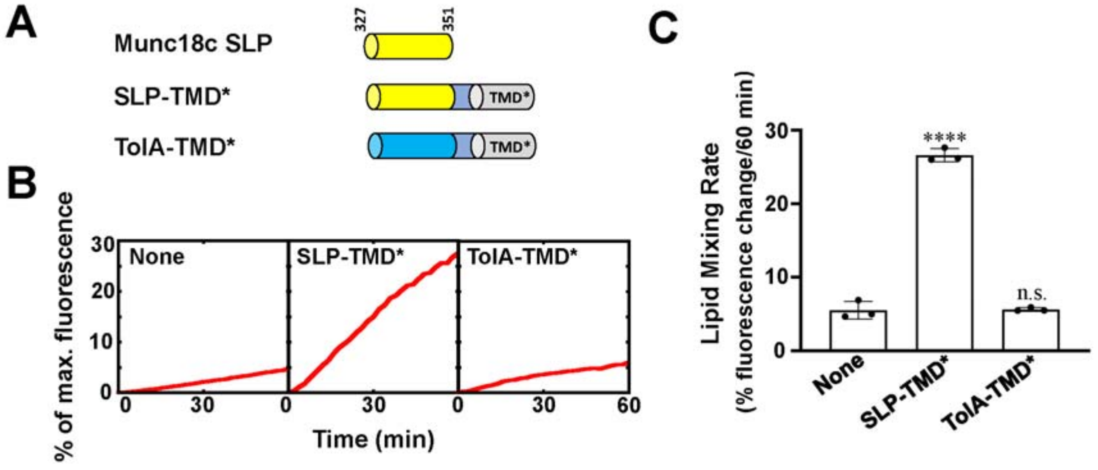

Figure S4. The membrane-anchored SLP activates SNARE-mediated membrane fusion independent of the anchor sequence. (A) Diagrams of Munc18c SLP and TolA with an engineered transmembrane domain (TMD*, from syntaxin4). **(B)** Lipid mixing of the reconstituted fusion reactions containing 5 µM t-SNAREs, 1.5 µM WT VAMP2, 100 mg/mL Ficoll 70, and 5 µM of the indicated chimeric protein. The fusion reactions were measured by a FRET-based lipid-mixing assay. **(C)** Lipid mixing rates of the reconstituted fusion reactions shown in B. Data are presented as percentage of fluorescence change per 60 min. Error bars indicate standard deviation. Data are presented as mean ± SD (n = 3 independent replicates). p Values were calculated using ordinary one-way ANOVA with Tukey's multiple comparisons test. n.s., $p > 0.05$. ****$p < 0.0001$.

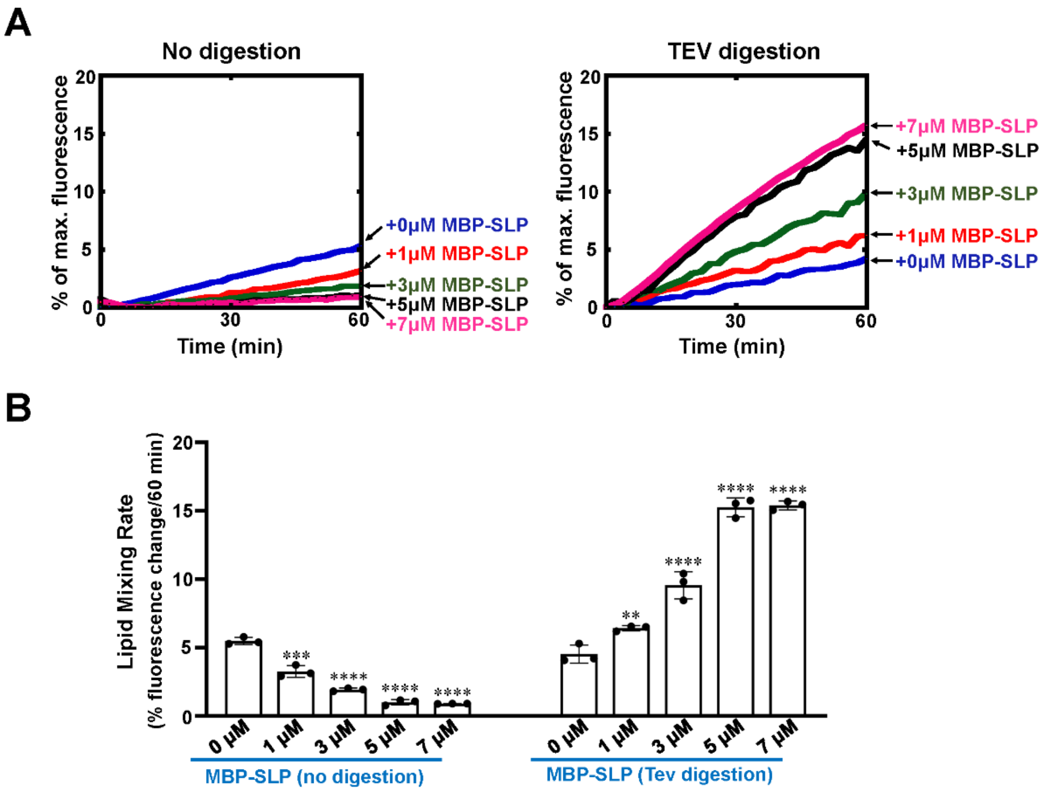

Figure S5. The soluble SLP concentration dependently activates SNARE-mediated membrane fusion in solution. (A) Lipid mixing of the reconstituted fusion reactions containing 5 µM t-SNAREs, the indicated concentration of MBP-SLP without (Left) or with (right) TEV protease, and 1.5 µM WT VAMP2. The fusion reactions were performed as in Figure 3B. All the reactions contained 100 mg/mL Ficoll 70. **(B)** Lipid mixing rates of the reconstituted fusion reactions shown in A. Data are presented as percentage of fluorescence change per 60 min. Error bars indicate standard deviation. Data are presented as mean ± SD (n = 3 independent replicates). p Values were calculated using two-way ANOVA with Tukey's multiple comparisons test. $^{**}p < 0.01$. $^{***}p < 0.001$. $^{****}p < 0.0001$.

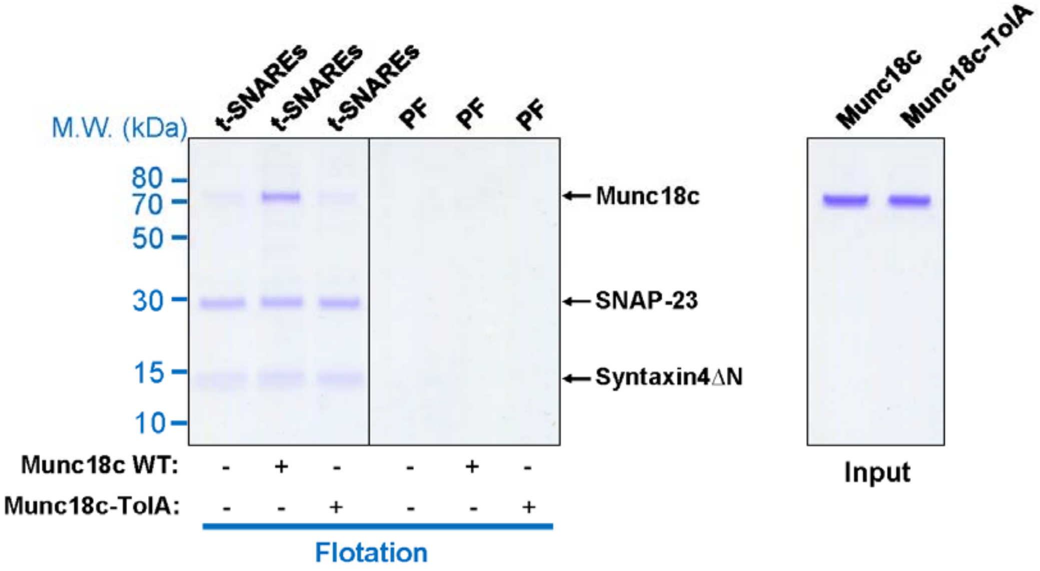

Figure S6. SLP is indispensable for Munc18c binding to the core domain of t-SNARE complexes. Coomassie blue-stained SDS/PAGE gel showing the binding of WT or mutant Munc18c to t-SNARE liposomes containing syntaxin4ΔN (the N-terminal aa. 1-172 was removed) and SNAP-23. PF, protein free. The liposomes were prepared with 100% PC. The reactions were carried out in the fusion reaction buffer. Each binding reaction contained 5 µM SNAREs and 5 µM indicated WT or mutant Munc18c protein.
